# Everything You Always Wanted to Know About Organoid-Based Models (and Never Dared to Ask)

**DOI:** 10.1016/j.jcmgh.2022.04.012

**Published:** 2022-05-25

**Authors:** Isabelle Hautefort, Martina Poletti, Diana Papp, Tamas Korcsmaros

**Affiliations:** 1 Earlham Institute, Organisms and Ecosystems Programme, Norwich, United Kingdom; 2 Quadram Institute Bioscience, Gut Microbes and Health Programme, Norwich, United Kingdom; 3 Imperial College London, Department of Metabolism, Digestion and Reproduction, London, United Kingdom

**Keywords:** Adult Stem Cells, Embryonic and Induced Pluripotent Stem Cells, Organoids, Hydrogels, Scaffolds, Microfluidics, Assembloids, *In Vitro* Models, aSC, adult stem cell, BMP, bone morphogenetic protein, ECM, extracellular matrix, ePSC, embryonic pluripotent stem cell, GIT, gastrointestinal tract, iPSC, induced pluripotent stem cell, RNAseq, RNA sequencing, WNT, Wingless and Int-1, 3D, 3-dimensional

## Abstract

Homeostatic functions of a living tissue, such as the gastrointestinal tract, rely on highly sophisticated and finely tuned cell-to-cell interactions. These crosstalks evolve and continuously are refined as the tissue develops and give rise to specialized cells performing general and tissue-specific functions. To study these systems, stem cell–based in vitro models, often called *organoids*, and non–stem cell–based primary cell aggregates (called spheroids) appeared just over a decade ago. These models still are evolving and gaining complexity, making them the state-of-the-art models for studying cellular crosstalk in the gastrointestinal tract, and to investigate digestive pathologies, such as inflammatory bowel disease, colorectal cancer, and liver diseases. However, the use of organoid- or spheroid-based models to recapitulate in vitro the highly complex structure of *in vivo* tissue remains challenging, and mainly restricted to expert developmental cell biologists. Here, we condense the founding knowledge and key literature information that scientists adopting the organoid technology for the first time need to consider when using these models for novel biological questions. We also include information that current organoid/spheroid users could use to add to increase the complexity to their existing models. We highlight the current and prospective evolution of these models through bridging stem cell biology with biomaterial and scaffold engineering research areas. Linking these complementary fields will increase the in vitro mimicry of *in vivo* tissue, and potentially lead to more successful translational biomedical applications. Deepening our understanding of the nature and dynamic fine-tuning of intercellular crosstalks will enable identifying novel signaling targets for new or repurposed therapeutics used in many multifactorial diseases.

**Summary**

Although revolutionary and increasingly used, organoids remain a challenging model for new users. In this review, we provide a general introduction for improving the accessibility to these models. We highlight areas for cross-disciplinary collaboration with biomaterial, tissue engineering, and nanofabrication sciences to broaden the application of organoids.

The sophistication and functioning complexity of all different organs in human beings are fascinating and yet so challenging to accurately define and investigate. Decoding the complex molecular and cellular interactions taking place in each organ, and how they malfunction in diseases, is instrumental to the progress of biomedical research and eventually to personalized medicine. Previously established *in vitro* models (cell lines, primary cell cultures) were either too simplified or not translatable to human beings and had limitations in recapitulating the different cell types and their interactions. Scientists have had to re-explore embryology and tissue development to devise and develop novel stem cell–based *in vitro* models that allow studying the mechanism of the vast range of interactions taking place within an organ in health and disease.

## The Beauty and Complexity of Tissues/Organs

The complexity of a functional organ resides mainly in the fact that all its cells sense, adapt, and respond to their immediate and distant environments. In the gastrointestinal tract (GIT), this includes not only external factors (eg, diet, microbes^1^, ^2^, ^3^) but also neighboring cells, cells from other tissues within the same or from distant organs.^1^^,^^4^, ^5^, ^6^, ^7^, ^8^ For example, the different cell types of the intestinal epithelium (eg, enterocytes, enteroendocrine cells, goblet cells) communicate with luminal or mucosa-associated microbes from the resident gut microbiota or with pathogens during infection.^9^, ^10^, ^11^ Intestinal epithelial cells also interact with each other and with tissue types from the intestine, such as the underlying mesenchyme,^12^, ^13^, ^14^ the gut-associated innate and adaptive immune system,^15^, ^16^, ^17^ the enteric nervous system,^13^^,^^18^, ^19^, ^20^ or even distant organs such as the liver, the lungs, or the brain.^21^, ^22^, ^23^

Accumulating evidence highlights the importance of maintaining an equilibrium between the intercellular crosstalks through intricate and dynamic regulatory pathways.^24^, ^25^, ^26^ Complex mechanisms ensure such biological systems can cope with transient fluctuations in the environment. Yet, alterations of key regulatory mechanisms (including host genetics or environmental factors) dramatically impact the growth, differentiation, maturation, and functions of many cell types. Malfunction of specific or multiple epithelial cell types consequently impairs intercellular crosstalks and can lead to chronic diseases such as Inflammatory bowel disease.^27^, ^28^, ^29^

This review focuses on how recently established stem cell–based models recapitulate host cell–cell interactions. We summarize the intrinsic limitations and complementarity of the different models that scientists should bear in mind when developing novel experimental approaches. In this review, we only briefly discuss the impact microbes have on intestinal cells and how this can be studied with stem cell–based models (for detailed descriptions, see reviews published elsewhere^30^, ^31^, ^32^).

## Stem Cell–Based Models: The Revolution for *In Vitro* Systems

Many factors and specific cell types are responsible for the maintenance of the stem cell niche, and for the differentiation of its progeny cells. Several of these molecules and cells have been identified already, such as epidermal growth factor, Wingless and Int-1 (WNT), R-spondin, bone morphogenetic protein (BMP), as well as pericryptal myofibroblasts, mesenchymal cells, and processes such as autophagy.^33^, ^34^, ^35^ However, their exact roles remain to be mechanistically unraveled for each cell type of the tissue of interest such as the intestinal epithelium. Filling these knowledge gaps requires improving *in vitro* culture systems of primary cells, particularly stem cells. Grown from stem cells and necessitating extracellular matrix-like scaffolding and specific niche factors, 3-dimensional (3D) cellular structures, termed *organoids*, can be created. Organoids can self-renew and generate *in vitro* functional structures containing the cell types present in the tissue they model (eg, mini-guts, mini-brains).^36^, ^37^, ^38^ These organoid models have now widely revolutionized *in vitro* models to study health and disease.

## The Powerful yet Challenging Advances Brought by Organoid Models

### Two Main Classes of Stem Cells Can Be Used to Grow Organoids

All differentiated cell types within an organ derive from progenitor cells, themselves being progenies of stem cells. Stem cells play an essential role in embryonic development and in the maintenance of most parts of an organ (eg, in the GIT they are essential for rapid renewal of the epithelium). Stem cells have been studied for decades and most recently have been used to develop *in vitro* cultures of organoids with cell types that to date could not be cultured in a dish.^39^ There are 2 main routes to developing stem cell–based *in vitro* models, relying on 2 main classes of stem cells: adult stem cells (aSCs) that reside within certain fast renewing tissues such as the GIT epithelium, the lung alveoli cells or the skin epidermidis, and pluripotent stem cells (either embryonic pluripotent stem cells [ePSCs] or induced pluripotent stem cells [iPSCs]).

#### Adult stem cells

Adult stem cells are undifferentiated cells naturally capable of self-regenerating asymmetrically. They renew themselves and produce progenitor cells that will proliferate and differentiate into all of the functional cell types normally residing in the tissue from which they derive.^40^, ^41^, ^42^ aSCs can be cultured *in vitro* to generate heterotypic 3D organoid structures, containing all or most of the different cell types normally present in the tissue of origin. aSC-derived organoids can be generated from healthy or diseased patient tissue samples,^43^, ^44^, ^45^ and animal models.^42^^,^^46^ Organoids can be maintained in culture for a long time through repeated passaging, during which they will maintain stable genetic and epigenetic signatures. During life, organs such as the GIT are exposed to different environmental signals (various microbes, food, antibiotics and general medications, inflammatory events, surgery), which will result in epigenetic modifications (eg, methylations, histone DNA packaging) within individual cells including stem cells.^47^ Although nongenetic, these modifications will be heritable by the daughter cells during mitosis, impacting gene expression in differentiated progeny cells. Hence, organoids derived from tissue of the similar organ or genetic background but carrying different epigenetic profiles will behave differently, reflecting the differences in the original donors.^47^, ^48^, ^49^, ^50^, ^51^ As a result, organoids generated from different host backgrounds (eg, diseased vs control patient-derived) will allow interrogating the role of epigenetic signatures on cellular functions and, thus, on cell–cell interactions taking place in these multicellular structures in health and disease.^50^, ^51^, ^52^

Depending on the source of stem cells used (adult, embryonic, or induced pluripotent), organoids can contain 1 or more tissue types. For instance, aSC-derived organoids established from intestinal crypt-derived stem cells will contain only epithelial cells. This type of organoid is ideal for achieving a simplified system, yet these models often lack the presence of underlying cells (eg, immune, mesenchyme, enteric nervous systems) and therefore will only reflect a limited part of the interactions staged in the whole intestinal system. To overcome this limitation, scientists have attempted growing cellular spheres derived from embedded minced tissue in an air–liquid interface culture system, successfully obtaining aSC-derived epithelial cells surrounded by a robust mesenchyme and stromal environment.^53^^,^^54^ Yet, this alternative culture method of aSCs requires a solid expertise of ex vivo tissue culture methodologies and cannot be the primary choice for new users of organoid models. In addition, aSCs are a scarce cell population in some tissues/organs, which sometimes makes their harvest challenging or impossible, and often necessitates the use of an alternative source of stem cells such as PSCs.

#### Embryonic and induced PSCs

Tissues that either do not contain easily culturable stem cells, or are not easily accessible for stem cells to be collected (eg, brain), also can be cultured as organoids from pluripotent stem cells (either ePSCs or iPSCs).^55^^,^^56^ ePSCs are the naturally present stem cells in an embryo, while iPSCs require first reprogramming of existing cells such as fibroblasts into PSCs. All PSCs are self-renewing cells that first derive into the 3 primary germ layers: ectoderm, endoderm, and mesoderm germ. In a second step, these germ layers will produce all cell types existing in the body.^57^, ^58^, ^59^ As a result, PSC-derived organoids can include more than 1 tissue type and neighboring cells to the tissue of interest (eg, the mesenchyme), opening the door to *in vitro* reproduction of many more *in vivo* intercellular interactions than aSC-derived organoids would allow.^57^^,^^60^ However, mastering the right time-dependent modifications of the culture conditions of these cells to obtain the correct germ layer, and subsequently guide its evolution through all correct developmental stages to result in the required organ-modeling organoids, is extremely difficult, making these models accessible to only specialized laboratories.^61^^,^^62^

**Figure 1:**
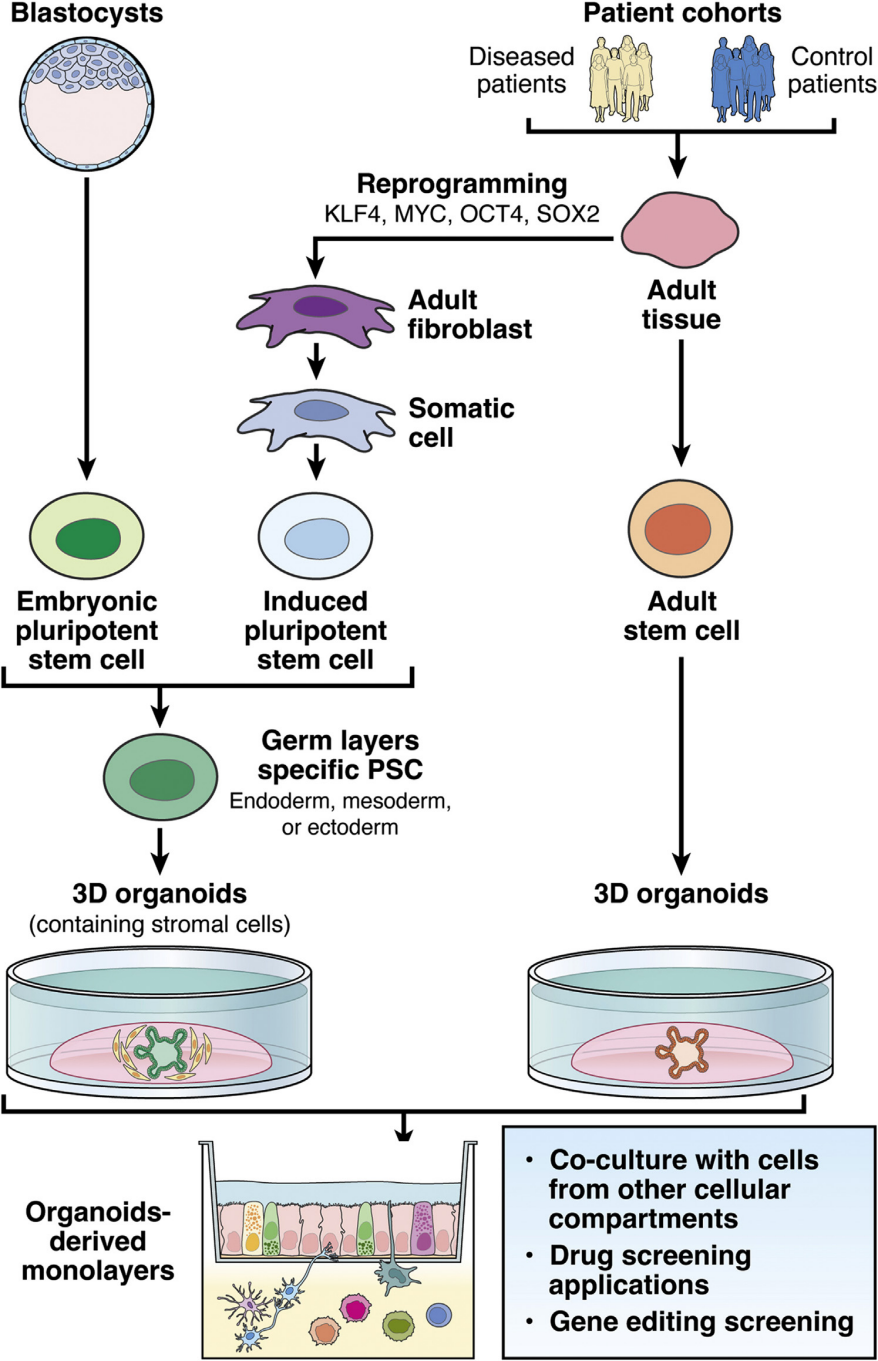
**Example of different formats of stem cell–derived intestinal organoid cultures.** Stem cells can be obtained from embryonic blastocysts or generated from adult tissue (biopsy specimens of diseased or healthy patients). aSCs (orange) can be used immediately to grow tissue-specific organoids. Embryonic or reprogrammed induced stem cells first need to develop into somatic cells (darker blue) and then the relevant germ layer (endoderm, mesoderm, or ectoderm; green) before being grown in tissue-specific organoids. Organoids then can be used directly to screen drugs or microbial metabolites in a patient-specific manner, or co-cultured with nonepithelial cells thought to interact with epithelial cells *in vivo*, either as 3D structures or as monolayers. To test the role of specific genetic determinants, organoids also can be genetically edited first and then used for compound screening or co-cultured with other cells containing different cell types (eg, secretory cells in green, yellow, and magenta, or absorptive in pink). KLF4, Kruppel-like factor 4; MYC, Protooncogene MYC protein; OCT4, (octamer-binding transcription factor 4; SOX2, SRY (sex determining region Y)-box 2.

In addition, ePSCs or iPSCs present some caveats in their accuracy to recapitulate important tissue traits in organoid culture. First, PSC-derived organoids show more embryonic features than aSC-derived organoids.^63^ Epigenetic signatures of iPSCs differ enormously from ePSCs because they can affect the reprogramming of fibroblasts into iPSCs.^58^ Therefore, ePSC- and iPSC-derived organoids present some distinctions in their potential use to model human genetic disorders (Figure 1).^64^ Although presenting fetal features, PSC-derived organoids can quickly gain adult maturation when first transplanted for kidney organoids, for example,^65^^,^^66^ as well as intestine, liver, pancreas, and retina organoids, as recently discussed.^67^

Finally, one major drawback of all stem cell– (aSC-, ePSC-, or iPSC-) derived *in vitro* models is that they might not always represent the region/part of the organ investigated. For example, PSC-derived intestinal organoid methods often would lead to the culture of small intestinal organoids instead of other intestinal regions (colon, cecum),^57^^,^^68^ limiting the range of applications of this model. This problem mainly was owing to the lack of deep knowledge on colonic tissue development until a few years ago.^57^^,^^68^ In recent years, the characterization of specific modulators of colonic signaling pathways, such as BMP, has allowed the development of iPSC-derived organoids into colonic tissue as well.^60^ Despite the limitations or technical challenges associated with all stem cell–derived *in vitro* models, organoids remain the closest *in vitro* systems to *in vivo* conditions.

### Animal Model Vs Human Organoids, a Tricky Choice

When selecting the best system to investigate the complex crosstalk happening at the organ level, choosing the right model organism is crucial. When studying the GIT, murine intestinal organoids from small intestinal aSCs represent the most documented organoid model, thanks to its accessibility, the easiness of establishment starting from a single intestinal crypt stem cell,^42^ and the availability of a wide range of genetic backgrounds. These models have allowed scientists to interrogate the role of particular genes, signaling pathways, or processes in the epithelial homeostasis,^69^^,^^70^ and how they are affected in particular.^40^^,^^69^, ^70^, ^71^, ^72^, ^73^ However, major immunologic, physiologic, and nutritional differences exist in animal model–derived organoids compared with human models, impeding the immediate translation of the obtained findings to human beings.

The development of human organoids from aSCs, ePSCs, or iPSCs is addressing this gap, and facilitates the screening of novel molecules before moving to clinical trials with greater chance of success. Human organoids now are used to study many diseases, from genetic, infectious, chronic, or cancerous nature.^39^^,^^74^^,^^75^ Genetic engineering applied to the organoid technology allows correcting genetic alterations *in vitro* or screening for drugs that could revert a mutation that plays a key role in disease pathogenicity.^76^^,^^77^ Patient-derived organoid lines now are being generated locally and are becoming accessible to more researchers through designated biobanks (eg, the Hubrecht Organoid Technology (HUB) Biobank, Utrecht, The Netherlands, https://huborganoids.nl; UZ/KU Leuven Biobank, Leuven, Belgium; Discover Together Biobank, Cincinnati Children’s Hospital, Cincinnati, OH). Such biobanks are reducing the requirement for geographic proximity of clinical research institutions to organoid/stem cell–derived tissue research laboratories. Through these biobanks/biorepositories the correct ethical regulations are carefully defined and maintained.^78^^,^^79^ Organoid lines are very appealing models to study intercellular crosstalk in health and disease, and can be compared with respective data from stratified patient cohorts.^27^^,^^28^^,^^40^

Human *in vivo* data to compare data obtained from human organoids are very scarce and definitely not easy to obtain without very invasive approaches, and the only alternative for preliminary studies is based on mouse organoid models. Mouse organoid models therefore still present many advantages, and can complement what is obtained on human organoids. In particular, mouse organoids have allowed pioneering technological advances in the field that then could be adapted to human organoid models for several tissues such as the brain.^80^ Thus, both species' organoids present important advantages and limitations and it is essential that new users question which species they should go for, when considering using organoid models for their research.

### Organoids Can Be Used to Study Cell–Cell and Cell–Microbe Interactions

Certain microbes or cell populations are critical to modulating homeostatic functions of an organ of interest. Enabling the *in vitro* co-culture of these different microbes or cell types is an obvious approach to understand their role within an organ. 3D organoids present an inward polarity, with their luminal side trapped within the 3D structure, making any apical challenge difficult or requiring microinjection. Recently, protocols have been developed to culture 3D organoids with a reverse polarity, making the apical side accessible, thus enabling microbial challenge to be applied as they would be encountered *in vivo*.^81^^,^^82^ Yet, this organoid model still requires further validation. Apical out organoids tend to be skewed toward absorptive cell lineage and may not fully recapitulate the epithelial cell type diversity present in the gut. In addition, the yield of 3D organoid reversion will not always be 100%, leading to variably mixed organoid populations.^30^

Adaptation of the 3D model sometimes is needed to enable further development of organoid-based models. Organoids can be grown as monolayers using extracellular matrix (ECM) protein-coated transwell filter inserts on which organoid fragments are seeded and allowed to form a confluent monolayer and then differentiate.^30^^,^^83^ Such a method is referred to as the *mucosoid cultivation system*, which was first developed to model the human gastric mucosa *in vitro*.^84^ In this model, the cells are cultured at the air–liquid interface, which induces cellular polarization and mucus production, while reserving their regenerative capacity.^84^ Furthermore, mucosoid cultures also allow studying the behavior of cell types specific to the gastric epithelium, such as chief cells, that was not possible *in vitro* before.^85^ Monolayers subsequently can be challenged with relevant signaling mediators (eg, microbial/dietary compound), as recently reviewed.^30^^,^^86^ Culture of organoid cells in monolayers has the advantage of giving access to both the cell apical and basolateral sides.

Microbes (commensals, probiotics, or pathogens or their products) can be applied to the apical chambers and interact with the organoid-derived monolayers.^17^^,^^87^, ^88^, ^89^ Organoid-derived monolayers can be grown within microfluidics devices that add shear forces associated with medium flow and gut wall smooth muscle stretching to the epithelial monolayer, reproducing many of the mechanical forces found *in vivo*, resulting in better mimicking of the epithelial monolayer differentiation.^90^, ^91^, ^92^ Nevertheless, limitations of these systems include their cost, the need for specific handling skills, and their requirement of lots of starting materials, making experiments not always affordable by many scientists. In addition, they are not yet applicable to the co-culture of tissue deriving from differing germ layers. More technological development would be needed to culture thicker organoid-derived complex cellular structure with the physical properties provided by microfluidics systems.

Direct interaction with neighboring cells found *in vivo* also can be recapitulated, at least partially, *in vitro*, involving co-culturing organoids as monolayers or 3D structures with 2 or more different cell types (Figure 1). Various examples for such approaches are given in Table 1. Recently, co-culture of murine aSC-derived or human iPSC-derived intestinal organoids with innate lymphoid cells from the respective species showed the impact of immune cells on the microenvironment of the epithelium, and how their malfunction can contribute to disease development.^93^ Co-culturing organoids with other key cell populations from the same individual within patient cohorts therefore could inform scientists and clinicians about the source of variations in the studied interactions between patients. This could highlight signaling regulation differences between individuals who otherwise show the same disease-associated symptoms, allowing precision medicine by stratification of patients and application of more appropriate therapies.

**Table 1:** Examples of Culture Approaches From Organoids

Organ modeled	Based on				Complexity	Field of research	References
	aSCs	eSCs/iPSCs	Isolated primary cells	Cell line			
3D apical-in organoids in Matrigel or 2-dimensional monolayers							
Mouse intestine	Y		Y		3D intestinal enteroids with separately isolated intraepithelial lymphocytes	Temporal and spatial interaction of intraepithelial lymphocytes with the intestinal epithelium	^94^^a^
Human colorectal and lung tissue	Y				3D grown enteroids of CRC and lung tumors were used to stimulate PBMC derived from the same patients	Tumor-specific T-cell–based targeting at the level of the individual patient, as a way forward to personalized medicine	^95^
Mouse intestine	Y		Y		3D intestinal enteroids embedded with isolated lamina propria lymphocytes	Probiotic influence on the lamina propria lymphocyte–mediated stem cell repair and epithelial barrier integrity	^96^^a^
	Y		Y		Small intestinal crypts, myofibroblasts, and myoplexus-derived neuronal cells mixed and embedded in ECM	Role of stromal cells such as fibroblasts and neurons in the development of the intestinal stem cell niche	^97^^a^
Synthetic hydrogels							
Mouse intestine	Y				Hydrogel-embedded 3D enteroids	Cell differentiation and influence of ECM stiffness	^98^
	Y				Collagen-soaked foam	Stem cell biology, drug, screening, tissue engineering, as well as regenerative therapies	^99^
Human intestinal epithelial cells, monocyte cell lines, and primary neutrophils				Y	Degradable and nondegradable hydrogels in high-throughput format	Effect of dynamic matrices on neutrophil infiltration into organoids	^100^
Human intestine and endometrium	Y				ECMs with tunable biomolecular and biophysical properties	Effect of ECM on ISC expansion	^101^
Human small and large intestines	Y				Synthetic hydrogels cross-linked by thiol-Michael addition reactions	New highly reproducible material for expanding intestinal organoids consistently	^102^
Human intestine					Synthetic hydrogels allowing 3D human intestinal organoid culture without encapsulation	New highly reproducible material allowing direct exposure of cultured 3D organoids to a stimulus of interest Highly relevant for regenerative and translational medicine	^103^
Human and mouse intestines and innate lymphoid cells 1	Y	Y	Y		Co-culture of primary ILC1s with intestinal organoids in various low-polymer concentration hydrogels	Intestinal epithelial cell–ILC1 interactions and impact of ILC1 on the extracellular matrix of the organoid stem cell niche	^93^
Transwell filters							
Mouse intestine, stomach	Y		Y		ECM embedded myofibroblasts or myenteric plexus ENS cells underlying ECM embedded intestine or stomach enteroids on Transwell filters	Interactions of epithelium with myofibroblasts and nerves (identification of stem cell niche factors)	^97^^a^
Human intestine	Y		Y		Small and large intestinal enteroid monolayers on collagen-coated Transwell filters preseeded under the filter with PBMC-derived macrophages	Intestinal epithelial cell–macrophage interactions and innate immune responses to infection of enteroids by bacterial pathogens	^104^^a^
Mouse and human intestine	Y	Y		Y	Monolayer enteroid grown on Transwell filter until confluency and transferred to well containing adipocytes	Proinflammatory signaling between IECs and adipocytes independently of immune cells	^17^^a^
Heterotypic spheroids/aggregates							
Rat liver				Y	Isolated rat hepatocytes cultured as microspheres first and then coated with fibroblasts (cell line)	Influence of surrounding fibroblasts to the maintenance of hepatocyte function	^105^^a^
Human intestine		Y			Human iPSCs endoderm-derived intestinal organoids and ectoderm neural crest cell-derived neurospheres, grown separately first and then co-cultured as 3D spheroids encapsulated in ECM for up to 4 weeks For longer culture, graft of the spheroids in mouse kidney subcapsular space for up to 10 weeks	Recapitulation of the architecture, vascularization, and function of the intestine including the myenteric and submucosal ENS and functions (gut motility) Model development for gut motility defect–associated diseases (eg, Hirschsprung disease)	^106^
Mouse and human intestine	Y			Y	U-shaped microwell made of defined hydrogels formed as arrays in plates and seeded with mouse or human intestinal organoid-derived single cells	Provides homogeneous, reproducible organoid arrays in less time than normal culture methods for testing various treatments/exposures and application to high-throughput readouts	^107^
Mouse immune organoid (B-cell germinal center)			Y	Y	Mouse primary B cells and 3T3 fibroblast cell line separately grown and then mixed encapsulated in ECM 3D structure	Novel model for B-cell germinal center	^108^
Scaffolds, patterned surface, microfluidic systems							
Mouse intestine	Y				Monolayer on scaffold support	Organization, cell differentiation, gut physiology	^109^
Human intestine	Y				Tubular perfusable microfluidic and scaffold-guided system using a mixture of collagen (for stiffness) and Matrigel	Physiological recapitulation of tissue architecture to investigate gut infection disease	^110^
	Y				Scaffold and chemical gradients	Architectural development of the intestinal stem cell niche	^111^
	Y			Y	3D silk tubular scaffold with intestinal enteroids seeded in the luminal compartment of a tubular silk scaffold and myofibroblasts seeded within the silk scaffold	New experimental scaffold to support, *in vitro*, intestinal epithelial cell growth, polarization, and differentiation from intestinal aSCs	^112^
Human liver, kidney				Y	Organ-on-a-chip (microfluidic system)	Organ-specific physiology	^113^
			Y	Y	Degradable layered hydrogel microfibers in a microfluidic system of fibroblast cell line and primary hepatocytes	Model development for long culture maintenance of hepatic functions	^114^
Human hepatocytes and fibroblasts				Y	Microfluidic for high-density 3D striped co-culture in hydrogel with varying physicochemical properties	Development of patterned culture system in controllable and heterogeneous hydrogel sheets for several cell types	^115^

CRC, colorectal cancer; ENS, enteric nervous system; eSC, embryonic stem cell; IEC, intestinal epitehlial cell; ILC, innate lymphoid cell; ISC, intestinal stem cell; PBMC, peripheral blood mononuclear cell; 3T3, fibroblasts.

a Studies in which intercellular interactions were addressed at least superficially.

## What to Consider When Adapting Organoid-Based Models to Unexplored Research Fields

Diverse environmental triggers are instrumental in shaping the conditions required for multicellular structures to grow *in vitro*. Self-organization of some organoids, such as intestinal organoids, depends strongly on sensing diffusible or cell surface-exposed signaling molecules from surrounding cells. Other organ models require forced specific cell pattern/layering to mimic the organ of interest.^116^ Mechanical shear forces from fluid passing over cells or from pulling and pushing through muscle contraction (eg, intestinal peristaltism) also influence the accuracy of the model developed.^117^^,^^118^ It therefore is paramount for new and existing organoid model users to choose a model based on many known factors, such as the source and types of cells to include, the level of simplification achievable, the availability of growth condition reagents, the scale of the planned experiments, and the different readouts applicable to that model. Despite the clear overlap in many existing protocols, there is no universal approach and many of the following factors will need to be considered separately and also in synergy for developing the appropriate model and answer specific biological questions.

### Cell Proliferation, Differentiation, and Maturation Are Influenced by the Surrounding ECM and Cells

In living tissues, mesenchymal and epithelial cells produce different components of the ECM, generating a gradient of signaling mediators important for tuning different pathways involved in tissue assembly, wound healing, and tissue regeneration.^119^ These include molecules such as integrins, laminin, collagen, fibronectin, entactin, and glycosaminoglycans.^34^^,^^120^ These components or their concentrations are unique to the different organs^121^ or specific tissue region (upper or bottom parts of intestinal crypts).^122^

ECM-like products derived from living tissue (ie, Engelbreth–Holm–Swarm mouse sarcoma) such as Matrigel (Corning, Flintshire, UK) or Cultrex (Trevigen, Gaithersburg, MD) promote cell adhesion with high efficiency and have become the by-default material scientists use for most organoid cultures. However, these products are very expensive and are derived from natural extracts, preventing researchers from labeling organoid experiments as animal-free. Matrigel usually contains fewer proteins (7–12 mg/mL) than Cultrex (12–17 mg/mL), restricting its use to self-organizing multicellular structures such as organoids, while Cultrex also can be used for culturing spheroids composed of cells of different sources.^123^ Each of these ECM products presents high batch-to-batch variability, especially in their protein content, causing reproducibility issues in organoid culture if not monitored.^124^, ^125^, ^126^

Several research groups have developed a wide assortment of basal cell–matrix protein–containing hydrogels, reproducing certain tissue-specific properties (different protein isoforms for different parts of a tissue).^122^^,^^127^^,^^128^ Initially for organoid model experts, these alternative ECMs, of more defined compositions, offer much-improved reproducibility and versatility than animal-derived matrixes to accommodate diverse organ-mimicking organoid cultures (see Table 1 for examples).^103^^,^^129^ Some allow the ECM to evolve/degrade dynamically as the epithelial structures grow,^100^ some offer reduced stiffness,^102^ while others have tunable biomolecular and biophysical properties.^122^^,^^101^

These technical advances enable optimizing the organoid cell size and differentiation, thus broadening the range of readout approaches that can be applied to organoids, for example, testing drugs or other host cell–derived secreted factors on disease-modeling organoids (eg, immune mediators).^130^^,^^131^ It therefore is primordial to gather as much information as possible about the ECM biochemical (eg, composition, protein isoforms, signaling growth factors) and physical properties (eg, stiffness) appropriate for the tissue to be modeled. A few recent reviews have compiled advantageous characteristics about currently available animal tissue–derived, or synthetic hydrogels, in their ability to promote and sustain organoid culture.^132^, ^133^, ^134^

Neighboring cells also will be the source of regulatory compounds of stem cell progenies’ fate. These cells will be more diverse in PSC-derived organoid cultures, and therefore will provide many more of these compounds than when aSC-derived organoids are used, in which case those regulatory molecules have to be added to the culture medium. Paneth cells, located at the bottom of the small intestinal crypts, contribute to the provision of several factors such as WNT3, necessary for cell proliferation and maintaining the stem cell niche, transforming growth factor-β, TNF-β, to favor the development of secretory cells such as enteroendocrine cells, or epidermal growth factor receptor, EGFR, which influences the transit-amplifying cell population.^135^, ^136^, ^137^ In the colon, where typical Paneth cells are not present, intestinal mesenchymal cells and Reg4+ deep crypt secretory cells are alternative sources of stem cell niche factors.^138^^,^^139^ The combined and tightly regulated effect of these compounds modulate key pathways such as the WNT, Notch, Hedgehog, BMP, and ephrinB pathways. Acting on these pathways regulation maintains the stem cell niche, and permits progenitor cells to differentiate into their functional form.^34^ The surrounding cellular environment of intestinal stem cells also comprises cells such as myofibroblasts, fibroblasts, endothelial cells, neural cells, smooth muscle cells,^34^ and resident immune cells (eg, macrophages, dendritic cells, regulatory T cells) that overall modulate ECM composition and host epithelial responses.^35^^,^^140^^,^^141^ Alongside neighboring cells, deeper tissue cells such as the enteric nervous neurons/glial cells will secrete factors such as transforming growth factor-β, 15-deoxy-Δ12,14-prostaglandin J2, glial cell-derived neurotrophic factor, or S-nitrosoglutathione essential not only for gut motility but also for the survival of stem cells, differentiation of their progenies, and maintenance of the epithelial barrier functions.^142^^,^^143^ All of these interactions in organoid cultures will have to be taken into account when interpreting organoid-based generated data because some will not be reproduced in the model.

Intestinal organoids can self-organize independently of other cells. Yet, their maturation more accurately resembles that of *in vivo* tissue when different surrounding cell types are present. To achieve this, co-culturing organoids in the presence of other cells is necessary. Co-culture of organoids with nonepithelial cells such as peripheral blood mononuclear cell–derived dendritic cells, intestinal intraepithelial lymphocytes, or endothelial cells already has been performed by using existing systems that originally permit direct or indirect contact between different cell types.^83^^,^^94^, ^144^, ^145^ For that, cells derived from organoids can be grown as monolayers on filter Transwell devices and exposed to signaling molecules secreted by other cells or directly to those cells (Figure 1). Alternatively, culture of organoids with other cell types into 150- to 400-μm diameter heterotypic 3D structures has proven useful in the case of hair follicle, intestinal, or kidney organoids.^107^, ^146^, ^147^, ^148^ Co-culture systems reproducing *in vitro* the tissue-specific cell movement and migration within organoids also have been developed successfully.^94^ These co-culture systems are highly relevant to investigate the interactions of infiltrating cell types with organoid cells ^56^^,^^149^ (eg, proinflammatory cells and homeostatic cells) (Figure 1).

### Tissue Topology, Cell Positioning, and Mechanical Forces Impact on Cell Differentiation and Maturation

Among the factors influencing the development and homeostasis of an organ, the organ 3D architecture increasingly is recognized as important. The 3D architecture encompasses the respective positioning and the distribution of the different cell types within the tissue.^150^ Little is fully understood about what regulates the spatial resolution of what makes an organ a functional organ. This highlights how useful it is to recapitulate at least part of this 3D landscape in an *in vitro* model to understand how it contributes to regulating cell functions. Attention to the tissue topology, cell positioning, and the shear forces applied to them therefore has gained importance as a valuable strategy in the development of more accurate organoid models.

Successful strategies to co-culture different cell types have included aggregating cells on coated surfaces or, conversely, in rotating vessels to prevent their adherence to the vessel itself. In parallel, using special scaffold coating or co-encapsulating the cells into defined ECM-mimicking hydrogels remains a preferred and more controllable approach. These options allow studying the different factors that influence cell survival in 3D cellular structures, including organoids.^95^, ^151^, ^152^

The stem cell niche maintenance and development is influenced strongly by the tissue topology (eg, curvature of the underlying tissue), the biomechanics (eg, shear forces from smooth muscle contractions of the digestive tract), and the permanent circulation of luminal flow. *In vitro* control of these additional factors strongly strongly impact the degree of proliferation, polarization, and differentiation of the pluripotent stem cell–derived structures,^153^ and it is clear that simplification of such variables is inevitable in mechanistic studies. Recently, intestinal aSC-derived organoid models were used to show the regulatory roles that intracellular crowding of macromolecules and volumetric compression of the cells have on stem cells and progeny growth, in particular on key cellular pathways such as WNT/β-catenin signaling pathways, and therefore on the organoid growth.^154^ Spatiotemporal control of the microcellular environment therefore is important when studying the cell type–specific function homeostasis and the involved intercellular crosstalks.^147^^,^^155^^,^^110^

### Possible Adaptation of Novel Hydrogels and Scaffolds to Organoids and Other Cell Co-culture

**Figure 2:**
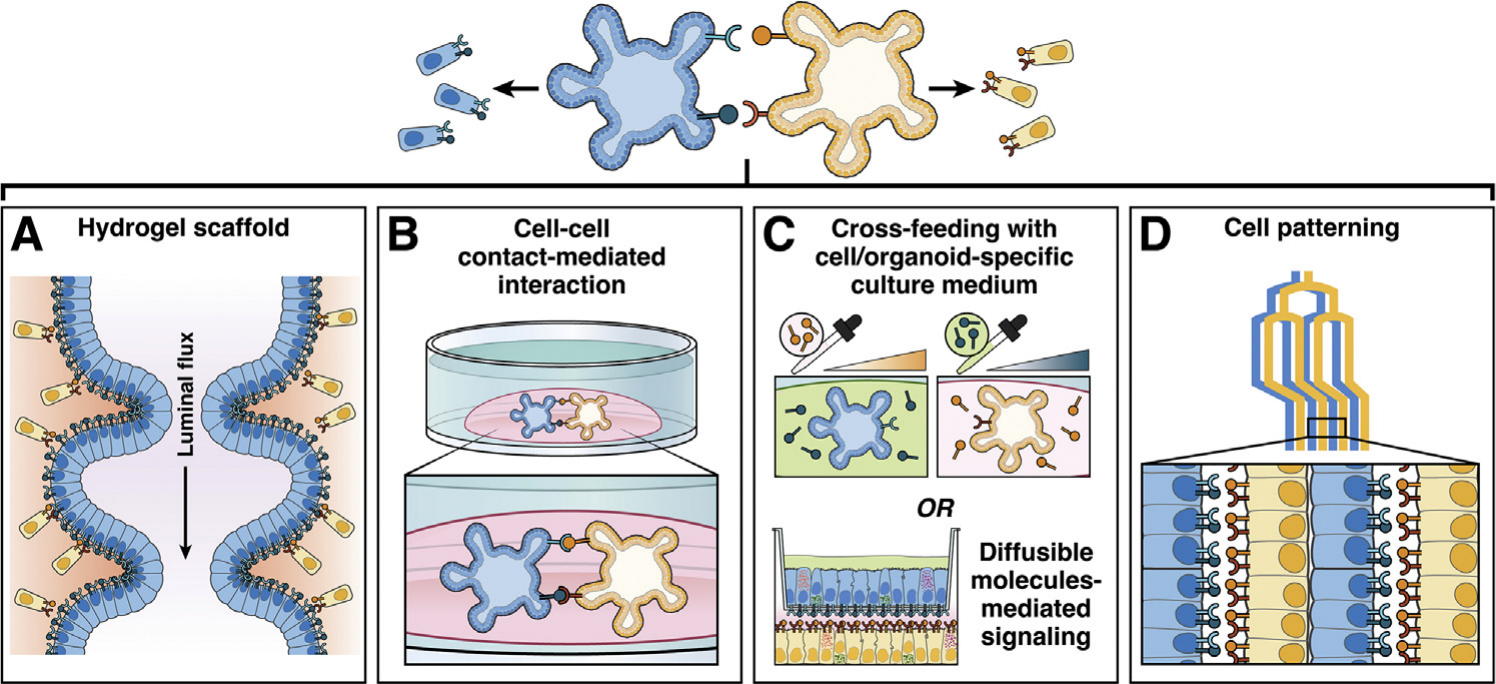
**Possible options for studying cell–cell communication.** (*A*) Mimicking tissue curvature and fluid circulation found in the organ will help generate organoids in open and perfusable systems. These setups could provide access to apical and basolateral sides for much longer periods of time than Transwells. This last yet promising option is still under development and will see the emergence of finely tunable model systems in the very near future. (*B*) Co-culture of organoids of interest within hydrogel domes that will self-organize allows investigation of contact-mediated interactions. (*C*) Cultures of organoids (in separate dishes or separated by Transwell filters as 3D or monolayers exposed to signaling compounds released in the culture medium. (*D*) In models in which cell differentiation into the mature cell type investigated depends on specific cell types alternating positions, microfluidics and gelation of organoid cells facilitates studying cell–cell interactions between matured cell types.

Considering the high level of versatility of classic co-culture systems, similar strategies are being adapted to organoid culture systems. Cells interacting *in vivo* can be first cultured separately *in vitro* before being seeded together (Figure 2*A*). Either Transwell filters or patterning scaffolds can be used for this purpose, the latter shows selective affinity toward specific cell types (Figure 2, Table 1).^156^

For instance, an interlocking comb-like silicon system was developed that already allows direct contact between 2 cell types in co-culture, as well as testing sustained short-diffusion range between cell types.^157^ Such an approach could facilitate studying contact-mediated or diffusible signaling taking place between cell types of interest in health and disease using diseased and control patient–derived organoids. For longer diffusion range, traditional Transwell co-culture of 3D organoids or organoid-derived monolayers with predicted interacting cells or their culture medium can help understand the role of secreted signaling factors in cell–cell crosstalk (Figure 2*B*).

Similarly, the pattern and layering of different cell types is of prime importance to better recapitulate cell–cell interactions, offering more control of the proliferation rate and differentiation state of the resulting organoid cells (Figure 2*C*). These microenvironmental signals will dictate how well the culture of organoids reflect the cell assembly and organization observed in the tissue of origin.^158^ Some technologies use magnetic nanoparticles and micromagnetic forces to help position different cell types, obtaining a more accurate cellular arrangement when studying their interactions.^159^ Similarly, different materials such as synthetic polymers can be used as scaffolds to control the levels of homotypic or heterotypic cell interactions in *in vitro* models.^160^, ^161^, ^162^ These materials first are modified and necessitate conjugation with bioactive molecules to permit their interactions with living cells.^101^

Engineering biomaterials can involve, for example: (1) scaffolds with *in* *vivo*–mimicking curvature (Figure 2*D*),^163^ (2) perfusable systems for supplying nutrients and oxygens to the complex 3D cell structures,^92^ and (3) defined hydrogels and relevant cell layering/positioning for mimicking *in vivo* intercellular crosstalks.^110^^,^^164^ Degradable hydrogel microfibers have been developed for layering and co-culturing mouse primary hepatocytes with fibroblasts, but could be adapted to stem cell–derived organoids too.^114^ Such structured hydrogels are particularly relevant in case of tissues, the functions of which cannot be recapitulated easily *in vitro* without specific layering or patterning (eg, tubular) architecture. Often, the self-organization conditions necessary for *in vivo* mimicking of that tissue have not been fully identified yet, impeding further advances in the translational fields of tissue bioengineering, repair, and/or replacement. Recent progress in biomaterial sciences offers *in vitro* systems that help specify the geometry of organoid-generating structures of defined shape, size, and cell distributions. Localized softening of hydrogels helps predict and control the geometry of murine intestinal organoids.^165^

Technical limitations, however, still are restricting the possibility to conduct longitudinal studies and explore the full differentiation of these heterotypic and quite large cellular structures. For example, classic ECM-embedded intestinal organoid cultures do not include a functional vascularization system. The larger the 3D structure is, the more limited oxygen supply becomes in the central part of the organoids, leading to hypoxia and exacerbated cell necrosis. To date, one possible way to culture organoids for a prolonged length of time is their xenograft to a living animal model tissue. This allows vascularization, that is, oxygenation of the organoids from the animal circulation system (eg, in the kidney subcapsular space,^106^ or peritoneal cavity in the mouse model,^166^ or the chicken chorioallantoic membrane^167^). Recently, an *in vitro* method was proposed using a patterned tubular matrix to grow organoids that self-arrange into an epithelial monolayer with crypt and villi regions (Figure 2*D*).^110^^,^^168^ This system allows perfusion of primary cell monolayers and culture of them for several weeks without hypoxia-induced damages.^110^ In the future, additional cell types could be included in the hydrogel scaffold of such models to investigate, for example, epithelial/immune cell interactions.

### Time: An Overlooked Parameter to Consider in Organoid-Based Models

One major breakthrough associated with organoid-based models is the ability to culture them over time. The generation of well-defined culture conditions for aSCs, ePSCs, and iPSCs has permitted organoids to be expanded indefinitely and to cryopreserve these organoid lines for future use.^169^^,^^170^ Organoid cultures were shown to maintain a high *in vitro* stability over time compared with the biopsy or tissue sample of origin,^46^^,^^128^^,^^171^^,^^172^ revolutionizing the use of *in vitro* primary cell-based models,^171^ and allowing us to move away from mouse models. The ability to expand organoid lines to relatively high passage numbers has enabled their use for high-throughput multi-omics technologies, producing transcriptomics, proteomics, metabolomics, and lipidomics data sets that can be explored to better unravel the various interactions taking place in a tissue.^69^^,^^173^, ^174^, ^175^, ^176^

## Outlook and Perspectives

### Current Challenges

Despite the great advances made in reproducing *in vitro* the *in vivo* chemical and cellular microenvironment, very few studies have produced mechanistic explanations of how organoids can mimic maturation, differentiation, and function of the different cell types found *in vivo*. As stem cell–based models, organoids have originated from embryology and developmental biology research, most progress is restricted to these research areas,^177^, ^178^, ^179^ leaving adult tissue function, repair, and homeostasis lagging behind. Furthermore, advanced understanding of fully formed and functioning organs is slowed down by the lack of native stromal cells, muscle cells, neurons and glial cells, blood vessels, and immune cells in organoid models, limiting the translation of organoid models to biomedical applications.

Equally important, applying organoid culture protocols to samples that originated from diseased tissue to recapitulate a disease phenotype is much more challenging than for healthy tissue (eg, tissue too damaged or containing high levels of apoptosis-triggering compounds).^28^ Access to improved reagents such as defined hydrogels is not yet widely accessible and remains the privilege of expert groups. Finally, the high financial cost associated with the development of sophisticated models is holding back the adoption of these models by many research groups. As a result, most detailed intercellular interaction studies still are based on simple models (Table 1). Still, a lot remains to be exploited from these evolving model systems for the generalized use of these models and the validation of mechanistic studies.

At last, choosing a relevant model strictly depends on the exact scientific question asked, hence, all different possible approaches should be considered while having that in mind.^180^ For example, is the studied disease monogenic or are there many genetic factors to control?^181^ Although mechanistic studies might require highly complex models, the screening of drugs or microbial products may be best performed in simpler models compatible with high-throughput formats.

Scientists embarking on the use of these promising models should acknowledge that the organoid technology is still in its infancy. Different ways to improve controllability and reproducibility of this technology should be pursued based on the specific scientific question asked. Additional parameters will need adding subsequently to control the cellular complexity,^31^ tissue geometry,^182^ and cellular patterning and layering of the modeled tissue/organ.^28^^,^^73^^,^^183^

### Novel Directions for Organoid Models

Currently, improved models are emerging from bridging stem cell research with biomaterial and bioengineering research fields in an attempt to replicate cellular pattern, tissue curvature, heterotypic diversity, shear forces from fluid flux, and neighboring cell movements. The next generation of organoid models are likely to contain most of the essential cell types present in an organ (eg, nerves, stroma, immune cells). They also will be developed following the concept of narrative engineering,^184^ that is, recapitulate the chronological changes (biochemical, mechanical, and physiological environment) as they would occur *in vivo*.

Once the various factors mentioned earlier become controllable, harmonized and standardized organoid-based models will be used by a larger part of the scientific community, providing the costs are reduced as well. Several studies already have provided lists of markers to check for a differentiation state of epithelial organoid cells co-cultured with nonepithelial cells (eg, intestinal organoids with cells from the enteric nervous system).^31^^,^^87^^,^^185^ Selected differentiation factors can be added to the culture to promote growth of specific cells that have not been cultured successfully *in vitro* from stem cells (eg, Receptor activator of nuclear factor kappa-B ligand, RANKL for generating microfold cells in intestinal organoids^186^). iPSC aggregates were shown to grow differently in the presence of different factors, other cells, or scaffolds; the core region of such structures remains very stable, while the peripheral parts respond more strongly to environmental changes.^187^

Standardization of organoid expansion alongside generation of stable organoid lines will form reliable tools for drug screening using high-throughput readouts (eg, single-cell RNA sequencing [RNAseq], Assay for Transposase-Accessible Chromatin using sequencing, ATAC sequencing, bisulfite sequencing, spatially resolved RNAseq, proteomics, and bioimaging).^45^^,^^188^, ^189^, ^190^ Recently, a multiplex single-cell analysis pipeline was developed on organoids co-cultured with fibroblast and leukocytes to establish post-translational modification signaling networks that can be altered in diseases.^191^ For example, growing organoids from patient-derived stem cell aggregates in preformed U-shaped microcavities imprinted in the hydrogel achieves highly homogenous cultures, both in size and maturation level. In addition, in this high-throughput single organoid model, cells will be positioned on the same Z plane, thus facilitating the automated live bioimaging screening of various drugs for the development of personalized medicine.^36^^,^^107^ These recent advances are instrumental for the reproducibility of experiments among different research laboratories across the world. Harmonizing these approaches at an international level will enable the successful translational biomedical applications for global pharmaceutical and biomedical companies/hospitals.^192^

Structural and mechanical scaffolds mimicking the microenvironment of the epithelial cells now are being developed,^124^ and will increase the capability of organoid cells to self-organize following layering or pattern that is important for those cells to fully mature and function as they would *in vivo* (Table 1, Figures 2 ). It now is foreseeable to combine organoid models of different organs into assembloids to study further intracellular interactions between different body systems such as the lungs, heart, gut, and nervous system.^193^^,^^194^ The tissue engineering research field has been a great source of innovation for developing improved organoid models dedicated to basic or translational research.^195^ Recently, a human brain organoid model was developed that also harbors optic vesicles recapitulating key cell types involved in vision (eg, corneal epithelial and lens-like cells, retinal pigment epithelia^196^). Combining these advanced models as a multi-organ system could be the strategy to fully comprehend homeostatic or diseased living biosystems.^197^^,^^198^ It still remains challenging to reproduce *in vitro* different communication axes such as gut–brain and gut–lung because of the simplified architecture of organoids, and more complex models still are required.^199^

What microfluidics systems (eg, gut-on-a-chip; Emulate, Boston, MA; Mimetas, organoplate, Oegstgeest, The Netherlands; organ-on-a-chip; Harvard Wyss Institute, Boston, MA) have enabled more recently was to recreate separated compartments, with the nutrient-containing medium side (on the basolateral side of epithelial surfaces) and the apical side of the epithelial barrier (luminal side of epithelial surfaces).^90^^,^^164^^,^^113^ These systems still are under improvement, but already can be exploited to recreate *in vitro* organ-specific features such as epithelium exposure to circulating fluid and flow-associated shear forces.^200^^,^^201^ An increasing number of organs have been modeled using these systems as reviewed by Huh et al,^113^ suggesting that such microfluidic systems could integrate several interconnected devices, each modeling a different organ (human-on-chip concept). This not only can permit cultures to be maintained for a length of time during experimentation, but also has been shown to lead to better maturation of the different cell types (co-)cultured.^153^^,^^202^ These platforms, although still expensive, offer great reproducible performance conditions that are incredibly useful for bioimaging, particularly live imaging of structures such as organoids^203^, ^204^, ^205^ (Table 1).

In addition to the multiple platforms emerging for using organoid-based models, the increasing accessibility to gene editing technology (eg, clustered regularly interspaced short palindromic repeats associated protein 9, CRISPR-Cas9) will bring forward more advanced regenerative and personalized medicine.^206^^,^^207^ It now is possible to confirm the genetic association of a mutation with a disease phenotype and to bring back functioning gene alleles, thus homeostatic functions in defective tissue.^77^^,^^208^^,^^209^ In parallel, assay formats and readout technologies also have evolved and now have become applicable to organoid-based approaches (eg, single-cell RNAseq, in situ RNAseq, and high-content live bioimaging), enabling high-resolution and longitudinal studies. Such technologies definitely will complement the development of better disease organoid models, as well as the understanding of the different levels of interaction that regulate tissue homeostasis, fostering future therapeutic approaches in human and animal health.

## Conclusions

In the past 10 years, stem cell–based research has made a huge leap forward, benefiting a myriad of other sectors, creating unforeseen collaborations between research fields such as biomaterials, microfluidics, high-throughput live bioimaging, mathematical modeling, data sciences, cellular biology, and multi-omics. Several biotechnology companies now offer already-made reagents/media to grow organoids, or alternative compounds to make growth medium from individual components, allowing creating diverse culture conditions for expansion, differentiation, or screening of organoids. Different already-made hydrogels and scaffolds also can be purchased (eg, Stemcell Technologies, https://www.stemcell.com; Corning and Amsbio extracellular matrices, https://www.corning.com/emea/en.html and https://www.amsbio.com; Biotechne and Peprotech culture supplements, https://www.bio-techne.com and https://www.peprotech.com/gb^168^). Various microfluidic platforms already are available to grow cell monolayers from organoids and offer accessibility to both apical and basolateral sides of epithelial cell layers (eg, Emulate, https://emulatebio.com; the HUMIX system^200^). Nowadays, protocols and training courses on how to establish organoid cultures from tissue samples or pluripotent stem cells are available (eg, https://www.cambioscience.com/2020/08/24/on-demand-3d-cell-models-course; https://www.stemcell.com/products/product-types/training-and-education/intestinal-organoid-training.html; and https://www.thermofisher.com/uk/en/home/life-science/cell-culture/organoids-spheroids-3d-cell-culture.html).

The health and disease-associated malfunctions of the GIT in particular now are studied with highly complex organoid models. Co-culture systems allow the scientific community to test whether specific cell–cell interactions are impaired in disease as a cause or consequence of the disease pathogenesis. Access to patient-derived organoid lines, developed from longitudinally collected samples from the same donor, could help investigate the impact of epigenetic signatures, disease-associated single-nucleotide polymorphisms, and messenger RNA splicing variants on the modulation of gene expression within the same patient. Future metagenomic and metabolomics profiling of patients’ intestinal microbiota also could help using organoids to study how microbes can influence the host cell–cell crosstalks and their different levels of regulation. The midterm future of intestinal organoid models therefore is promising to broaden our understanding of digestive diseases in a patient-specific manner. The same is envisageable for other organs.

The links currently developing between biology, biomedicine, biomaterials, and biophysics research with biotechnologies is a remarkable international initiative. It will boost the development of more relevant, reproducible, and amenable models to study intercellular interactions and their role(s) in health and disease.^168^ Future organoid-based models will become a goldmine resource for understanding the development and function of tissues at cell type–specific levels and in a patient-specific manner, including metadata such as age, gender, and medical history. With those models becoming more reliable, clinical trials of biologics pretested on organoids hopefully will be accelerated and tissue reconstruction will be elaborated with direct applications in regenerative and personalized medicine.

Although many studies now have shown the value of organoid models, only a few studies successfully have applied this technology to show mechanistic understanding of intercellular crosstalk.^93^^,^^97^ Although key cellular players influencing development, maturation, and functioning of a tissue have been identified, the molecular mechanisms involved remain to be elucidated. Emergence of novel stem cell–derived *in vitro* models, applicable to high-throughput technologies and combined with computational data sciences and mathematical modeling, will see the generation of extremely valuable multi-omics data, finally allowing us to decipher the mechanisms involved in intercellular crosstalks that govern the homeostatic functioning of a living tissue/organ and its alterations in diseases.^210^, ^211^, ^212^ Developed in collaboration with clinicians, these models can be developed from patient tissue, allowing us to interrogate genetic factors involved in the dysregulation of key homeostatic functions, and to screen novel as well as repurpose existing therapeutics.^213^, ^214^, ^215^ This brings the scientific and clinical communities much closer to finding new cures to diseases, or to simply prevent these diseases from developing into their severe forms in susceptible individuals.

All interested scientists should feel invited and encouraged to join this ongoing experimental revolution. Starting simple and adding complexity to the models should be the strategy of scientists new to the field to gradually build on their and others’ findings. Testing different options and optimizing them first is a prerequisite for the development and use of sophisticated intracellular interaction models. The coming years will see intercellular crosstalk mechanisms being sketched at a much deeper resolution level, and witness the emergence of many applications of organoid technology to unforeseen fields of research.
